# Metal-Organic Framework of Lanthanoid Dinuclear Clusters Undergoes Slow Magnetic Relaxation

**DOI:** 10.3390/ma10010081

**Published:** 2017-01-20

**Authors:** Hikaru Iwami, Ryo Nakanishi, Yoji Horii, Keiichi Katoh, Brian K. Breedlove, Masahiro Yamashita

**Affiliations:** 1 Department of Chemistry, Graduate School of Science, Tohoku University, 6-3 Aza-Aoba, Aoba-ku, Sendai, Miyagi 980-8578, Japan; iwami@dc.tohoku.ac.jp (H.I.); horii@dc.tohoku.ac.jp (Y.H.); kkatoh@m.tohoku.ac.jp (K.K.), breedlove@m.tohoku.ac.jp (B.K.B.); 2 WPI Research Center, Advanced Institute for Materials Research, Tohoku University, 2-1-1 Katahira, Aoba-ku, Sendai 980-8577, Japan

**Keywords:** metal-organic frameworks, single-molecule magnet

## Abstract

Lanthanoid metal-organic frameworks (Ln-MOFs) can adopt a variety of new structures due to the large coordination numbers of Ln metal ions, and Ln-MOFs are expected to show new luminescence and magnetic properties due to the localized *f* electrons. In particular, some Ln metal ions, such as Dy(III) and Tb(III) ions, work as isolated quantum magnets when they have magnetic anisotropy. In this work, using 4,4′,4″-s-triazine-2,4,6-triyl-tribenzoic acid (H_3_TATB) as a ligand, two new Ln-MOFs, [Dy(TATB)(DMF)_2_] (**1**) and [Tb(TATB)(DMF)_2_] (**2**), were obtained. The Ln-MOFs contain Ln dinuclear clusters as secondary building units, and **1** underwent slow magnetic relaxation similar to single-molecule magnets.

## 1. Introduction

Lanthanoid (Ln) metal ions with magnetic anisotropy, such as Dy(III) and Tb(III) ions, can work as isolated quantum magnets [1], which in most cases, are single molecules called single-molecule magnets (SMMs). Ln SMMs exhibit functional properties, like magnetic bistability and quantum tunneling of the magnetization (QTM) [2,3,4,5,6], making them useful in high-density magnetic memory devices [7,8], quantum computing [9] and molecular spintronics [10,11,12]. However, in order to use them, it is important to control the coordination environment and tune spin-spin interactions to enhance the magnetic properties of the Ln ions. Thus, metal-organic frameworks (MOFs) are good candidates for a variety of applications because their structures and coordination environments can be controlled by carefully selecting the ligands [13,14]. In particular, since Ln ions can have large coordination numbers, MOFs can be designed to be flexible and to have sophisticated structures. For example, Dy(III) ions and 1,3,5-benzenetricarboxylate (BTC) have been reported to assemble into a MOF with a highly symmetrical structure, which undergoes reversible slow magnetic relaxation driven by sorption or desorption of terminal coordinated water and guest molecules [15]. The magnetic properties of Ln-MOFs can be enhanced by changing their structures.

In this work, we prepared Ln-MOFs containing the ligand 4,4′,4″-s-triazine-2,4,6-triyl-tribenzoic acid (H_3_TATB), which has an elongated BTC structure with a triazine unit, to control the structure and coordination environment of the Ln ions. New Ln-MOFs of Dy(III) and Tb(III) ions were obtained by using a solvothermal procedure, and their structures were determined by using single crystal X-ray diffraction analysis. The Ln-MOFs contained Ln dinuclear moieties as secondary building units. In addition, from magnetic susceptibility measurements, the Dy-MOF underwent slow magnetic relaxation like SMMs do.

## 2. Results and Discussion

### 2.1. Syntheses of Ln-MOFs

**Scheme 1:**
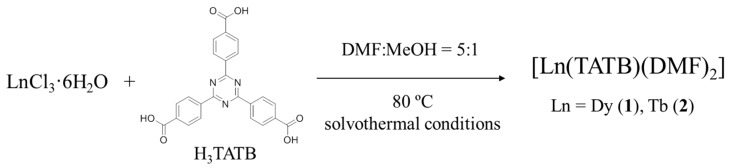
Synthesis of 1 and 2. Detailed procedures are given in the Materials and Methods section.

H_3_TATB was synthesized following reported procedures [16,17] and characterized by using ^1^H NMR and IR spectroscopies (Figures S1–S3). Then it was reacted with DyCl_3_·6H_2_O and TbCl_3_·6H_2_O under solvothermal conditions with N,N-dimethylformamide (DMF) and MeOH as solvents (Scheme 1). Ln-MOFs of Dy (**1**) and Tb (**2**) were obtained as colorless crystals (Figure S4), which were used for single-crystal X-ray diffraction analysis. The obtained crystals were washed three times by dispersing them in DMF, followed by ultrasonication, and were collected using ultracentrifugation. After drying by heating them in a vacuum, pure white powders were obtained, which were used for the magnetic susceptibility measurements.

### 2.2. Structural Analyses

**Figure 1:**
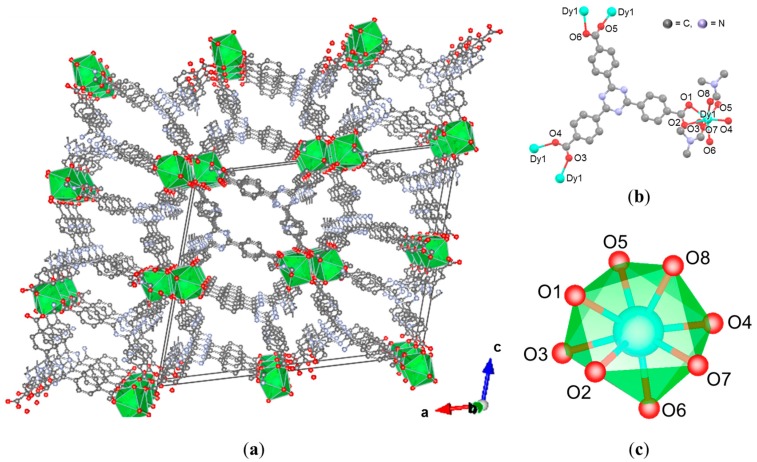
Drawings of (**a**) the crystal structures of lanthanoid metal-organic frameworks (Ln-MOFs) **1** and **2**; and (**b**) the asymmetric unit of **1**. Hydrogen atoms and crystal solvents are omitted for clarity; (**c**) Distorted square antiprism coordination polyhedron of the Dy(III) ions in **1**.

**Figure 2:**
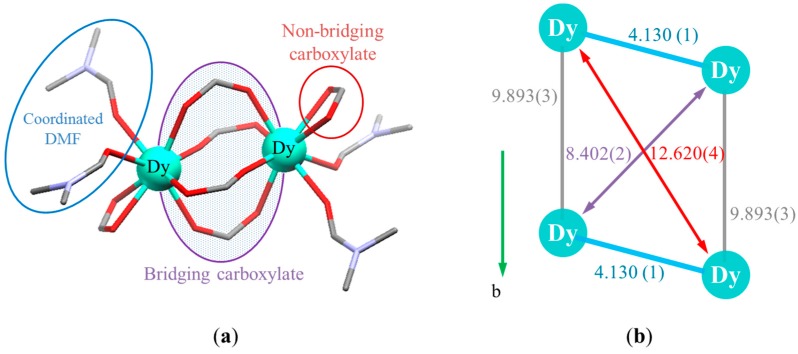
Drawings of (**a**) the Dy(III) dinuclear clusters in **1**; and (**b**) the Dy-Dy distances in Å.

From single-crystal X-ray diffraction studies at 110 K, **1** and **2** crystallized in the monoclinic space group *C*2/*c* and have similar structures, as shown in Figure 1a, with Ln dinuclear clusters as secondary building units. TATB^3−^ ligands intertwine without π-π stacking to form Ln-MOFs with small pores of less than 3 Å in diameter. The asymmetric units of **1** and **2** are shown in Figure 1b, Figures S5 and S6. The two Ln ions are bridged by four carboxylato groups, and the remaining coordination sites are coordinated by two non-bridging carboxylato groups and four DMF molecules. In **1**, each Dy(III) ion is in an 8-fold distorted square-antiprismatic coordination geometry (Figure 1c). In the 8-fold, local *D*_4d_ symmetry structure, the values of four dihedral angles are 5.6°, 6.3°, 57.3° and 43.5°. These values are slightly different with those of the ideal values for regular square-antiprismatic coordination geometry of 0°, 0°, 52.4° and 52.4°, which is due to the distortion. A drawing of the Dy-Dy unit and Dy-Dy distances of the dinuclear cluster in **1** are shown in Figure 2. The Dy-Dy distance in the dinuclear units in **1** was determined to be 4.13 Å, and the distance between dinuclear units was greater than 8.4 Å, indicating that Dy-Dy interactions in the dinuclear units were dominant in **1**. In the Tb(III) dinuclear units of **2**, an oxygen atom from two of the bridging carboxylato ligands bridge both Tb(III) ions, giving them a 9-fold distorted monocapped square-antiprismatic coordination geometry (Figure S7). Completing the 9-fold *C*_4h_ symmetry structure, a μ^2^ bridging O2 ligand acts as a cap, and the Dy-O2 bond length of 2.795(4) Å is much longer than others in the range of 2.316(3)–2.447(3) Å similar to the other Ln-Ln complexes, which have a 9-fold monocapped square-antiprismatic coordination geometries [18]. The structures of **1** and **2** are totally different from those of other reported Ln-MOFs containing H_3_TATB [16,19,20]. The reason for this difference is probably due to DMF, which can coordinate to the Ln ions. The crystal data for **1** and **2** are summarized in Table S1. We thought that these Ln-MOFs would exhibit SMM-like behaviour from the viewpoint of the structure because many Ln complexes exhibit SMM properties when they have square-antiprismatic [2,5,6,21,22] or monocapped square-antiprismatic [18,23,24] coordination geometries or when they have carboxylato-bridged dinuclear structures [18,25,26,27].

### 2.3. Alternating Current Magnetic Measurements

**Figure 3:**
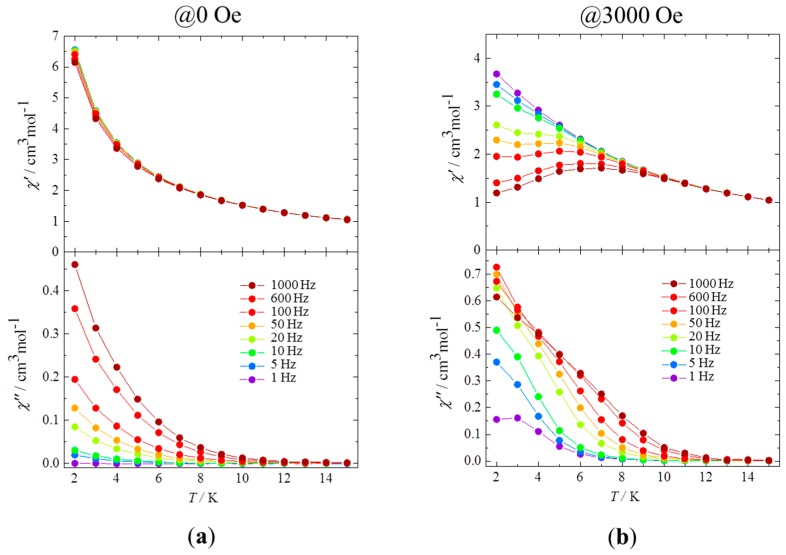
In-phase (χ′) and out-of-phase (χ″) AC magnetic susceptibilities vs. *T* plots for **1** in *H*_DC_ of (**a**) 0 and (**b**) 3000 Oe. The measurements were performed in an *H*_AC_ of 3 Oe in the frequency range of 1–1000 Hz.

To determine if **1** and **2** act as SMMs, alternating current (AC) magnetic measurements were performed. First, temperature dependent AC magnetic measurements were performed in high-*T* regions. Figure 3a,b shows plots of the temperature dependence of the in-phase (χ′) and out-of-phase (χ′′) signals of **1** with and without external magnetic field (*H*_DC_). In an *H*_DC_ of 0 Oe, the signals barely depended on the frequency (Figure S8). In an *H*_DC_ of 3000 Oe, the signals clearly depended on the frequency as shown in Figure 3, meaning that, in an *H*_DC_, the Dy(III) dinuclear clusters underwent slow magnetic relaxation, like SMMs. On the other hand, the χ values for **2** were not frequency dependent even in an applied field (Figure S9). This is probably due to the low symmetry of the 9-fold coordination sphere because Tb(III) ions need the higher symmetry of an 8-fold square-prism or square-antiprism geometry to have a magnetic easy axis [2,3,5,6].

**Figure 4:**
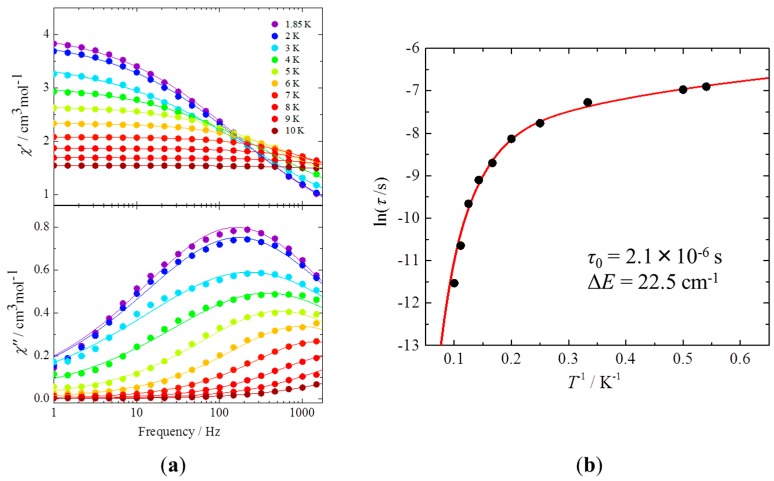
(**a**) Frequency dependence of χ′ and χ″ for **1** in an *H*_DC_ of 3000 Oe. The measurements were performed in *H*_AC_ of 3 Oe and *T* range of 10–1.85 K; (**b**) Arrhenius plot for **1** using the τ values obtained from AC magnetic susceptibility measurements at several *T*. The red line is a fit made by using Equation (1) with parameters *A* = 5.3 × 10^2^ s^–1^K^–1^ and *B* = 3.6 × 10^–5^ s^–1^.

Figure 4 shows the results of detailed dynamic magnetic studies on **1**. The χ values were measured in an *H*_DC_ of 3000 Oe. Both χ′ and χ′′ signals were clearly frequency dependent, and peak tops were observed for χ′′. From the plots, the relaxation time (τ) was determined by fitting the data with generalized Debye models (Equations (S1) and (S2)). The obtained τ values are plotted as a function of *T*^−1^ in Figure 4b. Considering that the Dy(III) ion (*J* = 15/2) is a Kramers ion, this plot can be fitted by using the following equation:

(1) $$\tau^{-1}=AT+BT^{9}+\tau_{0}^{-1}\;\exp\left(-\Delta E/k_{B}T\right)$$

where the first, second and third terms give the contributions of one-phonon direct processes, two-phonon Raman processes and thermally-activated Orbach processes, respectively [28,29,30,31]. τ_0_ is the pre-exponential factor, and Δ*E* is the activation energy. From Equation (1), τ_0_ for **1** was determined to be 2.1 × 10^–6^ s, and Δ*E* was determined to be 22.5 cm^–1^. These values are similar to those reported for some Dy(III)-based SMMs [15,18,22], although they are slightly lower due to the distorted coordination environment.

### 2.4. Direct Current Magnetic Properties and Calculations

**Figure 5:**
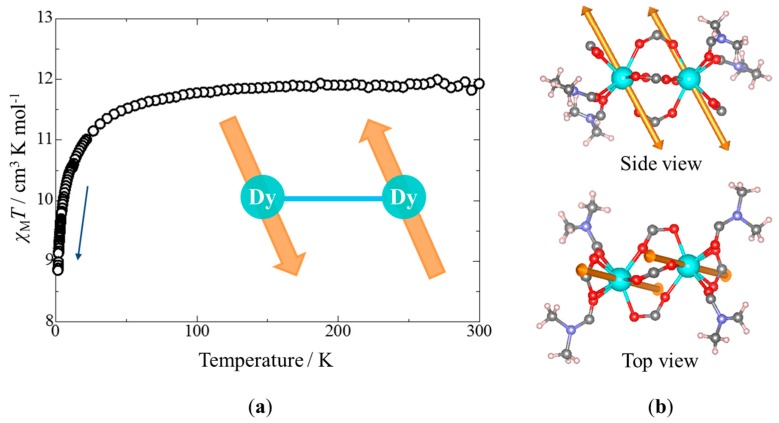
(**a**) χ_M_*T*-*T* plots for **1** and (**b**) drawings of the magnetic easy axes of dinuclear cluster in **1** calculated by using Magellan magnetic software. Inset of (**a**) is antiferromagnetic spin alignment of the dinuclear clusters in **1**.

To determine the Dy-Dy interactions in the dinuclear cluster of **1**, direct current (DC) static magnetic measurements were performed, and molar magnetic susceptibilities (χ_M_), which depended on *T* and *H*, were obtained. In χ_M_*T*-*T* plots, the χ_M_*T* value for **1** at 300 K was determined to be 11.92 cm^3^·K·mol^–1^, which is lower than that of an isolated Dy(III) ion of 14.2 cm^3^·K·mol^–1^ (Figure 5a). This is due to the presence of a large amount of solvent inside the pores, as determined by using thermogravimetric analyses (Figure S10). In the χ_M_*T*-*T* plots, the χ_M_*T* values decreased with a decrease in *T*, indicating that the Dy-Dy dinuclear cluster has anti-ferromagnetic interactions. From calculations using Magellan magnetic software [32], the Dy(III) ions in **1** were determined to have an anisotropy axis along the coordination direction of the non-bridging carboxylato groups (Figure 5b). Therefore, the spins in the Dy(III) dinuclear moieties are aligned oppositely along the anisotropy axes, as shown in the inset of Figure 5a. A Dy(III) dinuclear SMM having similar antiferromagnetic coupling, in which the antiferromagnetic exchange coupling between the two Dy(III) ions causes a temperature independent decrease in the χ′′ value in the low-*T* region and step-like features in the hysteresis loops, has been reported [33]. However, in our Dy(III) dinuclear cluster MOF, such features were not observed, as shown in Figure 3a,b and Figure S2 because the Dy-Dy distance in **1** (4.130 Å) is larger than that of the reported SMM (3.768 Å), meaning that the antiferromagnetic interactions are weakened. In addition, the weak interactions are reflected in the lack of clear hysteresis in the *M*-*H* curve for **1** (Figure S11). Because the structure of the MOF can be tuned precisely by changing ligands or introducing molecules into the pores, we believe that it is possible to control the Ln-Ln distance and enhance the magnetic properties of this type of Ln-MOFs.

## 3. Materials and Methods

### 3.1. General

All reagents were purchased from Wako chemicals (Osaka, Japan), Tokyo Chemical Industry (TCI) (Tokyo, Japan), and Sigma Aldrich (St. Louis, MO, USA) and used without further purification. Distilled water was obtained from an EYELA STILL ACE SA-2100E deionizer (Tokyo Rikakikai Co. Ltd., Tokyo, Japan). ^1^H NMR spectra were acquired on a 500 MHz Bruker AV-500 spectrometer (Billerica, MA, USA). IR measurements were acquired on a JASCO FT/IR-4200 spectrometer (Tokyo, Japan).

### 3.2. Synthesis of 4,4′,4″-s-Triazine-2,4,6-Triyl-Tribenzoic Acid (H_3_TATB)

Syntheses were performed following the literature [16,17]. Trifluoromethanesulfonic acid (3.0 mL) was added slowly to *p*-tolunitrile (1.5 mL, 7.3 mmol). The mixture was stirred for 18 h, poured on ice and neutralized with ammonia water. The precipitate was collected by filtration and then washed with water and acetone. Recrystallization in toluene gave 2,4,6-tri-*p*-tolyl-1,3,5-triazine as white crystals (2.43 g, yield: 94.9%). ^1^H NMR (500 MHz, CDCl_3_): δ 8.67 (d, *J* = 5.0 Hz, 6H), 7.39 (m, *J* = 5.0 Hz, 6H), 2.51 (s, 9H).

The obtained 2,4,6-tri-*p*-tolyl-1,3,5-triazine (1.54 g, 4.31 mmol) was put into a 300 mL three-necked flask, to which 90 mL of acetic acid and 3.0 mL of H_2_SO_4_ were added. Chromium oxide (3.93 g, 25.9 mmol) and 10 mL acetic anhydride were added with stirring while keeping the temperature below 50 °C. The resulting green-brown slurry was stirred overnight, poured into 100 mL of cold water, and the resulting solution was mixed well and filtered. The collected solid was washed with water and dissolved in 200 mL of a 2 N NaOH solution. After the unreacted starting material was removed by filtration, the solution was acidified with HCl to give crude product. Recrystallization from DMF gave pure product as a white solid (1.83 g, Yield: 96.5%). IR (cm^–1^): 3070 (m), 2665 (w), 2540 (w), 1702 (vs), 1584 (s), 1522 (vs), 1362 (s), 1289 (s), 1017 (m), 829 (m), 764 (s). ^1^H NMR (500 MHz, *d*_6_-DMSO): δ 8.22 (m, 6H), 8.88 (m, 6H), 12.9 (br, 3H).

### 3.3. Synthesis of [Dy(TATB)(DMF)_2_] (**1**)

DyCl_3_·6H_2_O (19 mg, 0.050 mmol) and H_3_TATB (21 mg, 0.050 mmol) were dissolved in a DMF/MeOH (10/2.0 mL) in a 20 mL vial at ambient temperature. The reaction mixture in a capped vial was heated in an oven at 80 °C for 3 day. Colorless crystals of **1** were obtained with a yield of 72.3% based on 1.0 mol of H_3_TATB.

### 3.4. Synthesis of [Tb(TATB)(DMF)_2_] (**2**)

Using the same procedure as that used for **1**, except using TbCl_3_·6H_2_O (19 g, 0.050 mmol) instead of DyCl_3_·6H_2_O, colorless crystals of **2** were obtained with a yield of 86.1% based on 1.0 mol of H_3_TATB.

### 3.5. X-ray Crystallography

Single-crystal X-ray analyses for **1** and **2** were carried out on a Rigaku Saturn 724+ CCD diffractometer (Rigaku Corporation, Tokyo, Japan) with graphite-monochromated Mo-Kα radiation (λ = 0.71069 Å) at *T* = 110 K, mounted on a loop rod coated with Paratone-N (Hampton Research Corp., Aliso Viejo, CA, USA). An empirical absorption correction based on azimuthal scans of several reflections was applied. The data were corrected for Lorentz and polarization effects. All non-hydrogen atoms were refined anisotropically using a least-squares method, and hydrogen atoms were fixed at calculated positions and refined using a riding model. SHELXL-2014/7 [34] was used for structure refinement, and the structure was expanded using Fourier techniques. The weighting scheme was based on counting statistics. Full-matrix least-squares refinements on *F*^2^ based on unique reflections with unweighted and weighted agreement factors of *R* = ∑||*F*_o_| − |*F*_c_||/∑|*F*_o_| (*I* > 2.00 σ(*I*)) and *wR* = [∑*w*(*F*_o_^2^ − *F*_c_^2^)^2^/∑*w*(*F*_o_^2^)^2^]^1/2^ were performed. Visualization and analysis of the crystal structures were simulated from the single crystal data by using Mercury 3.8 [35].

### 3.6. Thermogravimetric Analysis

Thermogravimetric analyses (TGA) were performed on a SHIMADZU DTG-60 (Kyoto, Japan) in a nitrogen flow using aluminum oxide powder as a standard material. Several milligrams of sample were put in an aluminum cell, and the cell was heated to 550 °C with a heating rate of 2 °C/min.

### 3.7. Magnetic Susceptibility Measurement

Magnetic susceptibility measurements were performed on a SQUID magnetometer (Quantum Design, model MPMS-XL SQUID magnetometer, San Diego, CA, USA). Samples were placed into gel capsules, and *n*-eicosane was added to fix the samples during the measurement. Temperature dependent DC measurements were recorded in an *H*_DC_ of 1000 Oe. *T* was changed from 300 K to 1.85 K with a sweep rate of 1 K/min. Field dependent DC measurements were performed at 1.85 K with changes in the *H*_DC_ as follows: 0 Oe → 50 kOe → –50 kOe → 50 kOe. AC measurements were recorded in an *H*_AC_ of 3 Oe in the frequency range of 1–1500 Hz, in the *T* range of 10–1.8 K, and in *H*_DC_ fields of 0 and 3000 Oe. Diamagnetic corrections for the molar magnetic susceptibilities were applied using Pascal’s constants.

## 4. Conclusions

In this work, we synthesized two Ln-MOFs, [Dy(TATB)(DMF)_2_] (**1**) and [Tb(TATB)(DMF)_2_] (**2**). The MOFs had similar structures containing lanthanoid dinuclear clusters as secondary building units. We found that **1** underwent slow magnetic relaxation similar to SMMs due to the Dy(III) ions having 8-fold square-antiprismatic coordination geometries, whereas **2** did not due to the 9-fold distorted, low symmetry coordination structure of the Tb(III) ions. Since it should be possible to change the coordinating DMF solvents to other molecules in these MOFs, we are currently studying how to best tune the structure and control their magnetic properties. Furthermore, we are preparing magnetically diluted MOFs by substituting some of the Dy(III) and Tb(III) ions with diamagnetic Y(III) ions, which will lead to a better understanding of their magnetic properties such as the contribution of the depopulation of sublevels of the ground J multiplet split by the CF. Because these MOFs have many advantages, such as the structural flexibility and high thermal stability, enhancement of their magnetic properties and use in applications, such as memory devices, are possible.

## Supplementary Materials

The following are available online at www.mdpi.com/1996-1944/10/1/81/s1. Figure S1: ^1^H NMR spectrum of 2,4,6-tri-*p*-tolyl-1,3,5-triazine in CDCl_3_ at 500 MHz on a Bruker AV-500 spectrometer; Figure S2: ^1^H NMR spectrum of 4,4′,4″-s-triazine-2,4,6-triyl-tribenzoic acid (H_3_TATB) in CDCl_3_ at 500 MHz on a Bruker AV-500 spectrometer; Figure S3: IR spectrum of H_3_TATB; Figure S4: Obtained transparent crystals of (a) **1** and (b) **2**; Figure S5: ORTEP drawing of asymmetric unit of **1** with thermal ellipsoid of 50% probability. Hydrogen atoms are omitted for clarity; Figure S6: ORTEP drawing of asymmetric unit of **2** with thermal ellipsoid of 50% probability. Hydrogen atoms are omitted for clarity; Figure S7: Drawings of (a) Tb(III) dinuclear clusters in **2**, and (b) their Tb-Tb distances. The unit of length is Å; Figure S8: Frequency dependences of the (a) χ′ and (b) χ′′ AC magnetic susceptibilities of **1** in an *H*_DC_ of 0 Oe. The measurements were performed in an *H*_AC_ of 3 Oe and *T* range of 10–1.85 K; Figure S9: Temperature dependences of the χ′ and χ′′ AC magnetic susceptibilities of **2** in *H*_DC_ of (a) 0 and (b) 3000 Oe. The measurements were performed in an *H*_AC_ of 3 Oe and *T* range of 10–1.85 K; Figure S10: Thermogravimetric analyses of **1** and **2**; Figure S10: Thermogravimetric analyses of **1** and **2**; Figure S11: Field dependence of the magnetization of **1** at 1.8 K. Slight hysteresis was observed. Table S1: Crystal structure parameters for **1** and **2**. Equations (S1) and (S2): Generalized Debye model.
