# Supplementary material for: Metal-Organic Framework of Lanthanoid Dinuclear Clusters Undergoes Slow Magnetic Relaxation
Source: Materials (Basel). 2017 Jan 20;10(1):81. doi: 10.3390/ma10010081 (PMC5344597; doi:10.3390/ma10010081)
Supplement: Supplementary file 1 [file materials-10-00081-s001.pdf]

Supplementary Materials: Metal-Organic Framework of Lanthanoid Dinuclear Clusters Undergoes Slow Magnetic Relaxation

Hikaru Iwami, Ryo Nakanishi, Yoji Horii, Keiichi Katoh, Brian K. Breedlove and Masahiro Yamashita

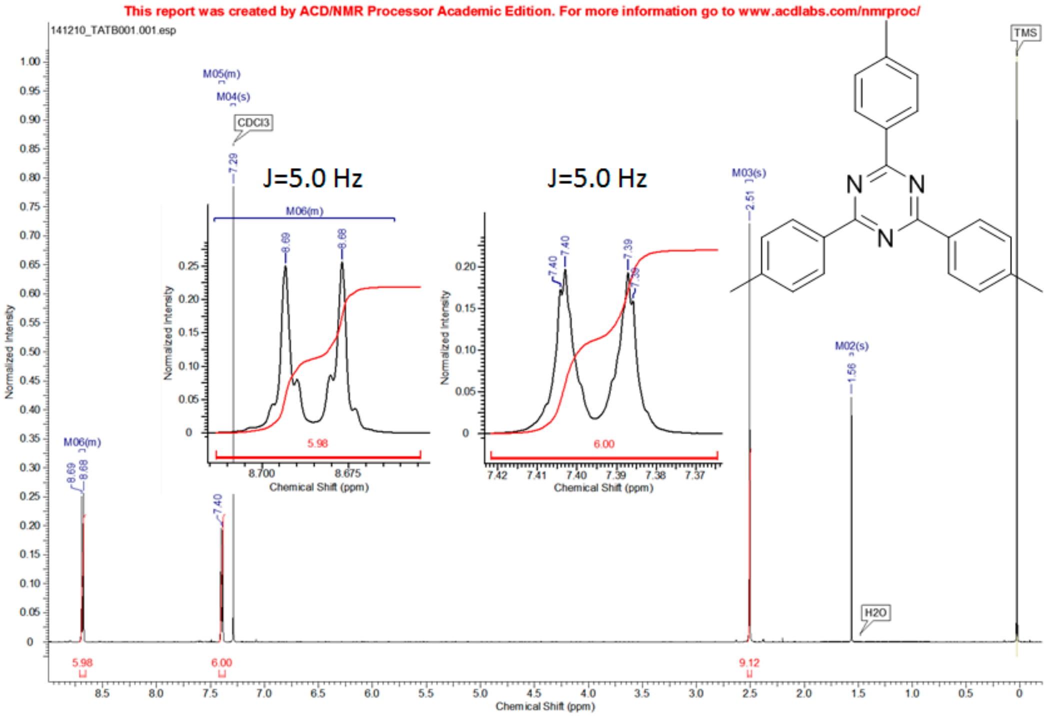

Figure S1. ^{1}H NMR spectrum of 2,4,6-tri-*p*-tolyl-1,3,5-triazine in $CDCl_3$ at 500 MHz on a Bruker AV-500 spectrometer.

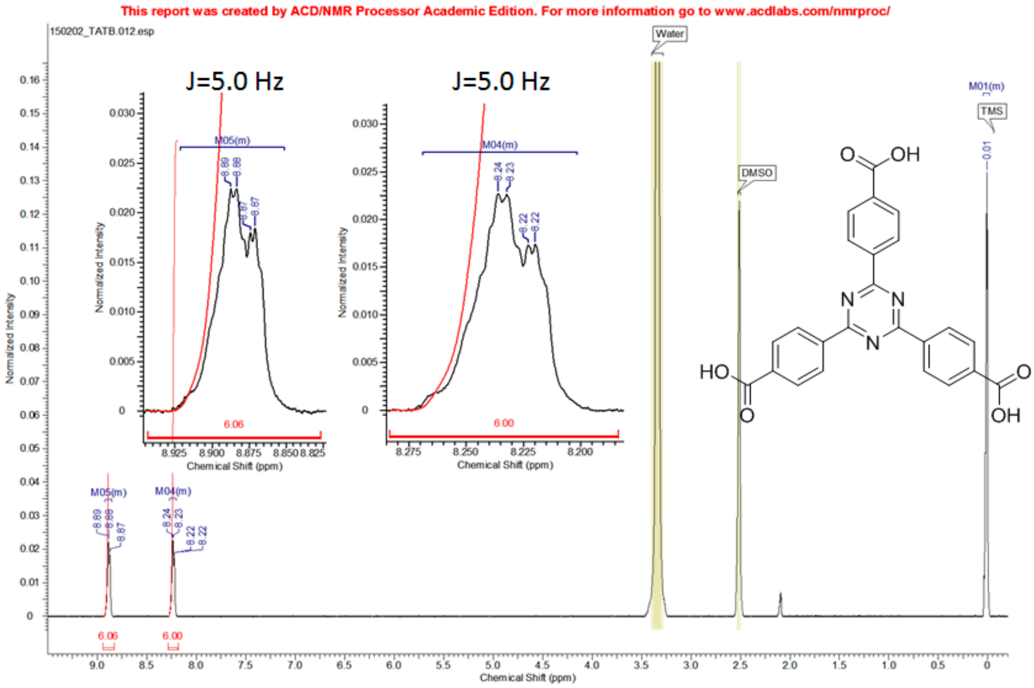

Figure S2. ^{1}H NMR spectrum of 4,4′,4″-s-triazine-2,4,6-triyl-tribenzoic acid (H_3TATB) in $CDCl_3$ at 500 MHz on a Bruker AV-500 spectrometer.

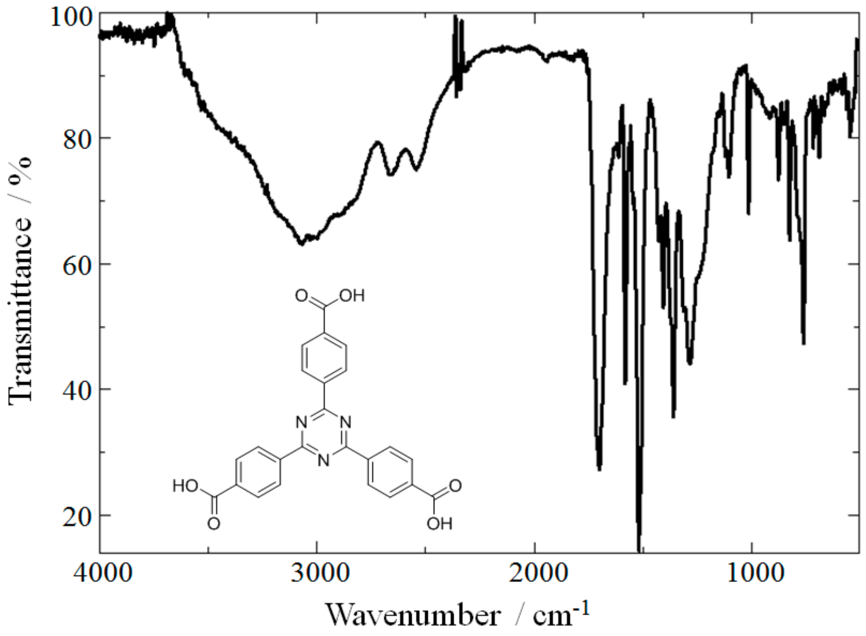

Figure S3. IR spectrum of H_3TATB.

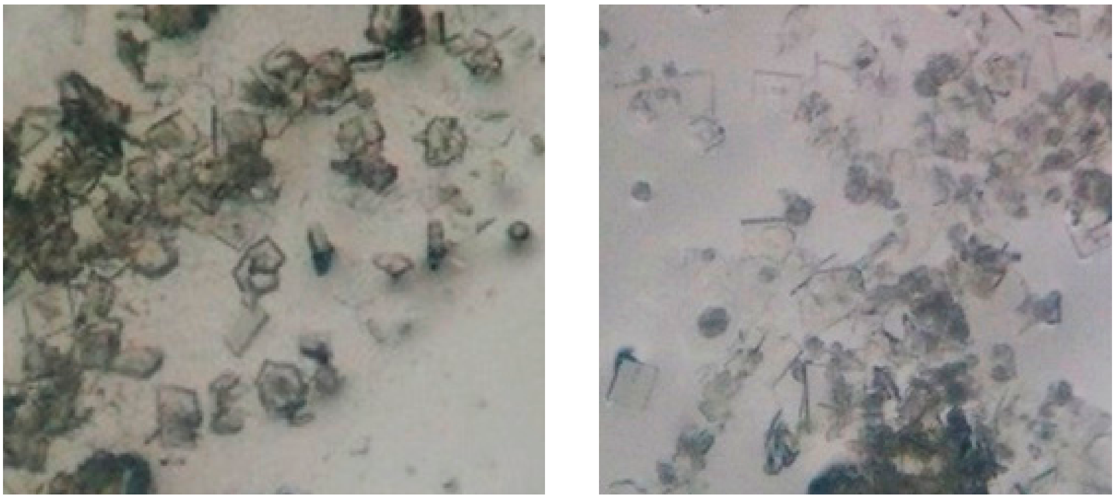

(**a**) (**b**)

Figure S4. Obtained transparent crystals of (**a**) **1** and (**b**) **2**.

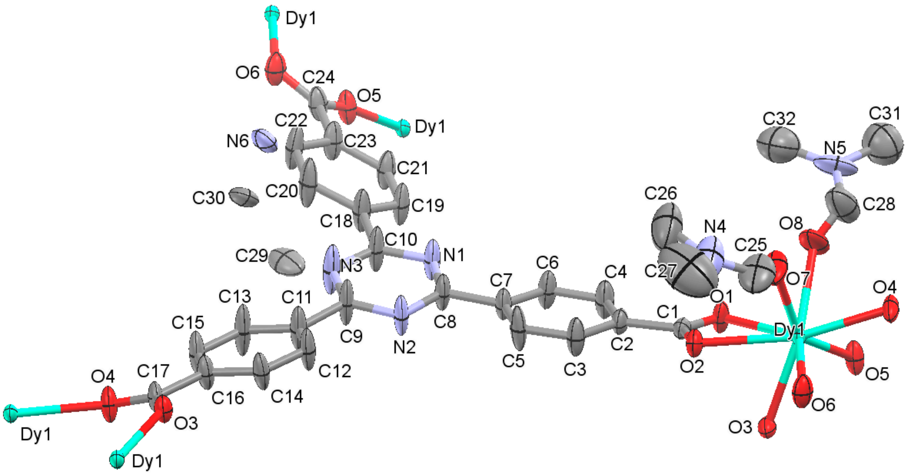

Figure S5. ORTEP drawing of asymmetric unit of **1** with thermal ellipsoid of 50% probability. Hydrogen atoms are omitted for clarity.

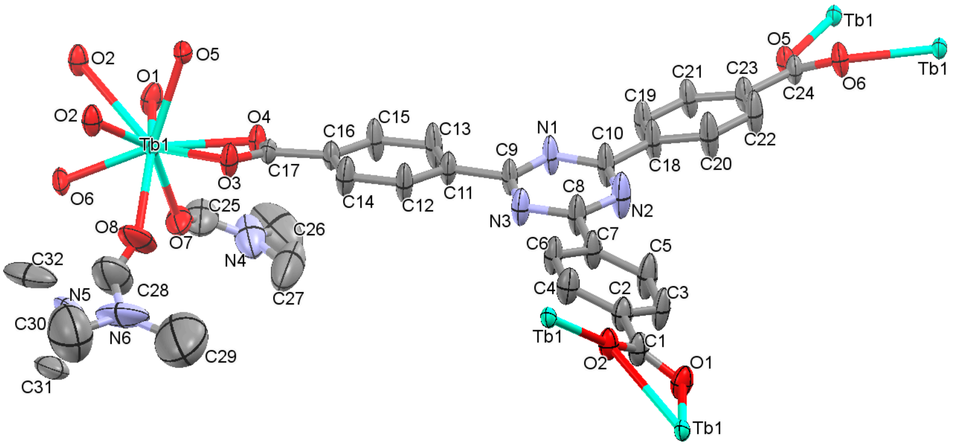

Figure S6. ORTEP drawing of asymmetric unit of **2** with thermal ellipsoid of 50% probability. Hydrogen atoms are omitted for clarity.

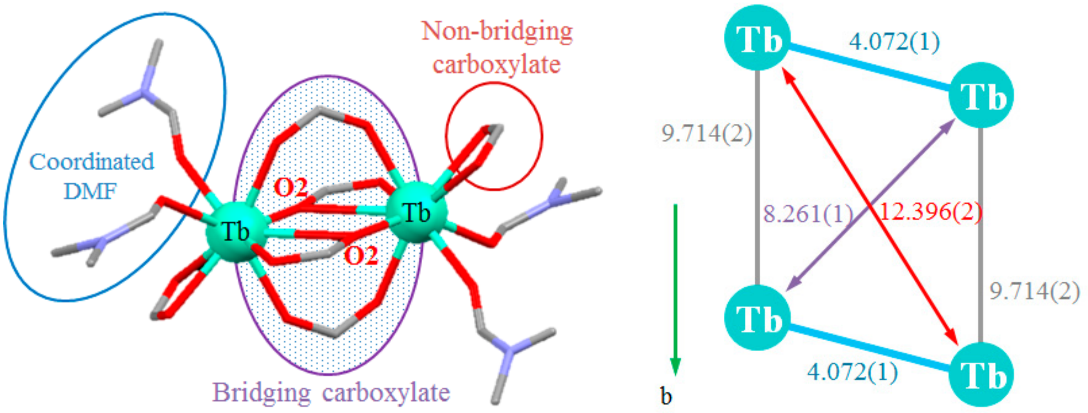

(**a**) (**b**)

Figure S7. Drawings of (**a**) Tb(III) dinuclear clusters in **2**; and (**b**) their Tb-Tb distances. The unit of length is Å.

Table S1. Crystal structure parameters for **1** and **2**.

Compounds	1 [Dy(TATB)(DMF)$_2$]	2 [Tb(TATB)(DMF)$_2$]
Formula	$C_{30}H_{26}N_5O_8Dy$	$C_{30}H_{26}N_5O_8Tb$
Formula mass/g·mol^{-1}	747.06	743.48
T/K	110	110
Crystal system	monoclinic	monoclinic
Space group	$C2/c$	$C2/c$
a/Å	33.138(10)	33.137(7)
b/Å	9.893(3)	9.7145(17)
c/Å	26.959(8)	27.314(6)
α/°	90	90
β/°	106.751(5)	107.642(3)
γ/°	90	90
V/Å^3	8463(4)	8379(3)
Z	8	8
R_1 [$I > 2s(I)$] [a]	0.0476	0.0461
ωR_2 (all) [b]	0.1328	0.1237
GoF on F^2	1.011	1.098
CCDC number	151,5211	1,516,111

$^{a}\, R_1 = \sum(F_0 - F_c)/\sum F_0;\ wR_2 = \sqrt{\sum[w(F_0^2 - F_c^2)]/\sum[w(F_0^2)^2]}$.

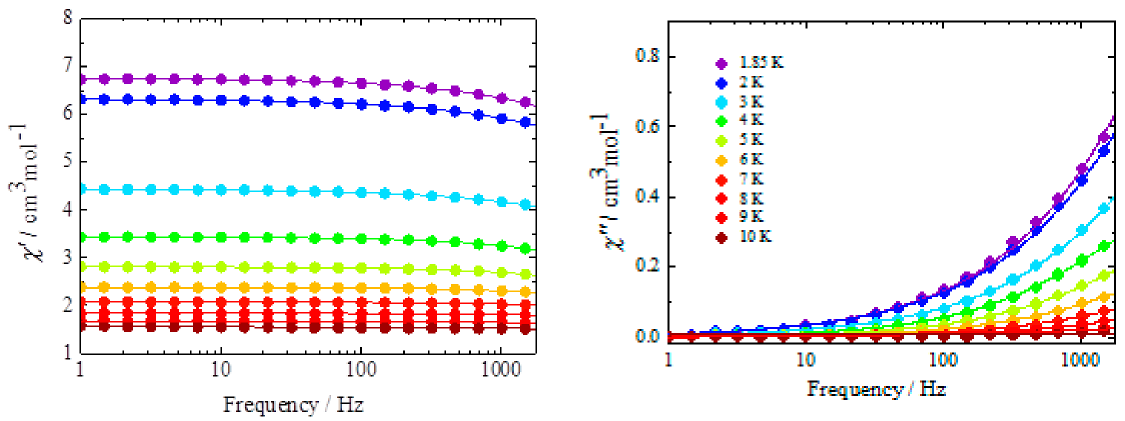

(**a**) (**b**)

Figure S8. Frequency dependences of the (**a**) χ′ and (**b**) χ″ AC magnetic susceptibilities of **1** in an *H*DC of 0 Oe. The measurements were performed in an *H*AC of 3 Oe and *T* range of 10–1.85 K.

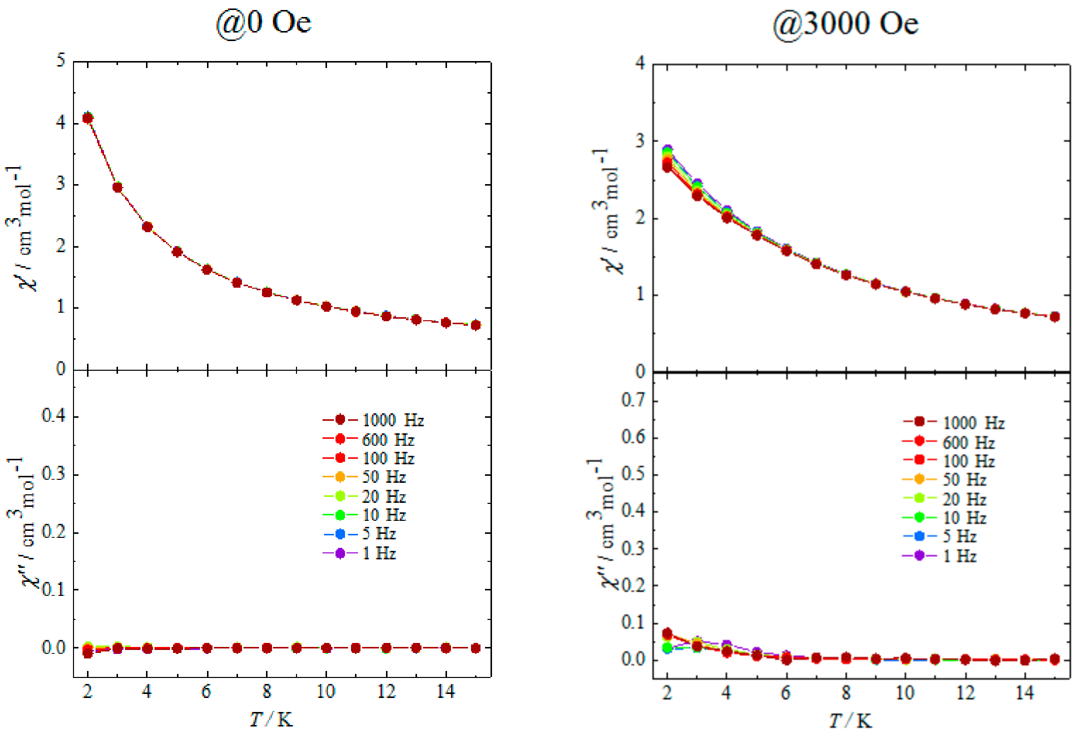

(**a**) (**b**)

Figure S9. Temperature dependences of the χ′ and χ″ AC magnetic susceptibilities of **2** in *H*DC of (**a**) 0 and (**b**) 3000 Oe. The measurements were performed in an *H*AC of 3 Oe and *T* range of 10–1.85 K.

The Generalized Debye Model (Equations (S1) and (S2))

$$\chi'(\omega) = \chi_S + (\chi_T - \chi_S)\frac{1 + (\omega\tau)^{1-\alpha}\sin(\pi\alpha/2)}{1 + 2(\omega\tau)^{1-\alpha}\sin(\pi\alpha/2) + (\omega\tau)^{2-2\alpha}} \quad \text{(S1)}$$

$$\chi''(\omega) = (\chi_T - \chi_S)\frac{(\omega\tau)^{1-\alpha}\cos(\pi\alpha/2)}{1 + 2(\omega\tau)^{1-\alpha}\sin(\pi\alpha/2) + (\omega\tau)^{2-2\alpha}} \quad \text{(S2)}$$

where χ_S is the adiabatic susceptibility, χ_T is the isothermal susceptibility, $\omega = 2\pi\upsilon$, (υ is the frequency) is the angular frequency, τ is the magnetization relaxation time, and α is the quantitative parameter for the width of the τ distribution.

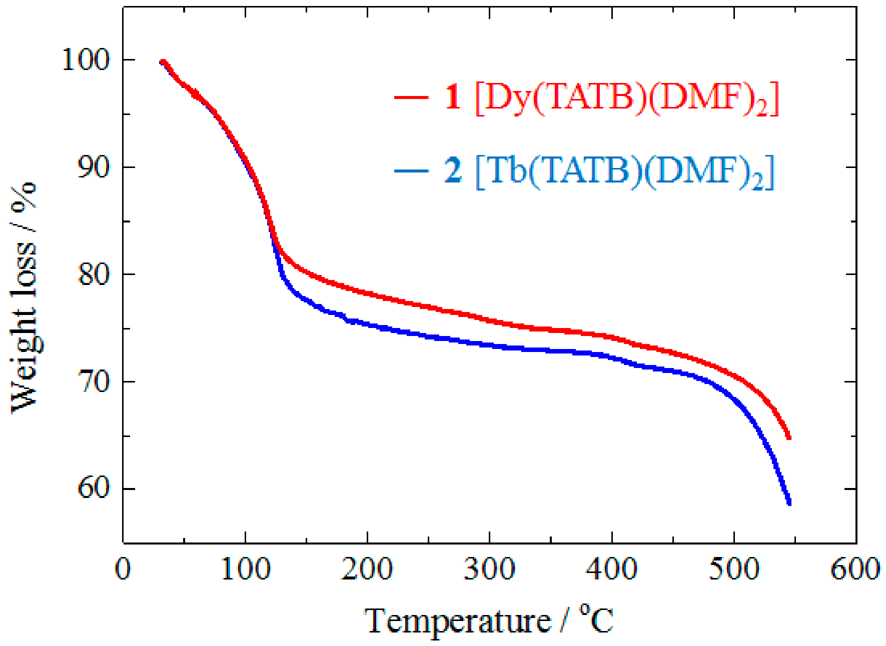

Figure S10. Thermogravimetric analyses of **1** and **2**.

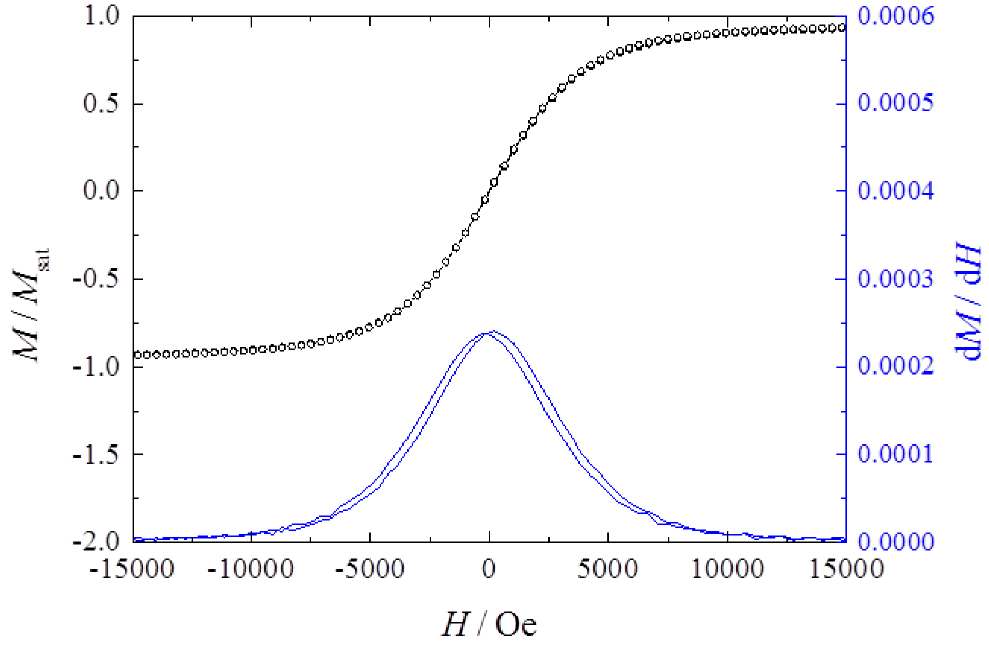

Figure S11. Field dependence of the magnetization of **1** at 1.8 K. Slight hysteresis was observed.
